# Kinetics of local and systemic immune cell responses in whirling disease infection and resistance in rainbow trout

**DOI:** 10.1186/s13071-019-3505-9

**Published:** 2019-05-21

**Authors:** Mona Saleh, Ruth Montero, Gokhlesh Kumar, Arun Sudhagar, Adina Friedl, Bernd Köllner, Mansour El-Matbouli

**Affiliations:** 1 Clinical Division of Fish Medicine, Department for Farm Animals and Veterinary Public Health, University of Veterinary Medicine Vienna, Vienna, Austria; 2 Institute of Immunology, Friedrich-Loeffler-Institut, Federal Research Institute for Animal Health, Südufer 10, 17493 Greifswald-Insel Riems, Germany

**Keywords:** *Myxobolus cerebralis*, Flow cytometry, Myeloid cells, B cells, T lymphocytes

## Abstract

**Background:**

Whirling disease (WD), caused by the myxozoan parasite *Myxobolus cerebralis*, is responsible for high mortalities in rainbow trout hatcheries and natural populations. To elucidate how resistant and susceptible rainbow trout strains respond to early invasion, a well-established model of WD was used to demonstrate the kinetics of local and systemic immune responses in two rainbow trout strains, the susceptible American Trout Lodge (TL) and the more resistant German Hofer strain (HO).

**Methods:**

Parasite load and cellular immune responses were compared across several time points after *M. cerebralis* exposure to elucidate the kinetics of immune cells in resistant and susceptible rainbow trout in response to early invasion. In the course of the 20 days following exposure, leukocyte kinetics was monitored by flow cytometry in the caudal fin (CF), head kidney (HK) and spleen (SP). For the analysis of the leukocyte composition, cells were stained using a set of monoclonal antibodies with known specificity for distinct subpopulations of rainbow trout leukocytes.

**Results:**

Experiments indicated general increases of CF, HK and SP myeloid cells, while decreases of B cells and T cells in the SP and HK were observed at several time points in the TL strain. On the other hand, in the HO strain, increases of T cells were dominant in CF, HK and SP at multiple time points. The differences between HO and TL were most distinct at 2, 4, 12 and 48 hours post-exposure (hpe) as well as at 4 days post-exposure (dpe), with the vast majority of innate immune response cells having higher values in the susceptible TL strain. Alteration of the leukocyte populations with augmented local cellular responses and excessive immune reactions likely lead to subsequent host tissue damage and supports parasite invasion and development in TL.

**Conclusions:**

The findings of this study highlight the significance of effective local and systemic immune reaction and indicate proper activation of T lymphocytes critical for host resistance during *M. cerebralis* infection. The present study provides insights into the cellular basis of protective immune responses against *M. cerebralis* and can help us to elucidate the mechanisms underlying the variation in resistance to WD.

## Background

Whirling disease (WD) is a debilitating disease of salmonids caused by the myxozoan parasite *Myxobolus cerebralis*, which evolved in Europe as a parasite of the brown trout (*Salmo trutta*). The disease was first discovered in rainbow trout (*Oncorhynchus mykiss*) imported from North America for the growing aquaculture industry in Europe in the late 1800s [1]. The introduction of the non-native rainbow trout into Europe led to the discovery of the disease. In the USA, *M. cerebralis* was first detected in 1958 in Pennsylvania, and since then WD has been confirmed in 25 US states. The disease has serious economic and ecological impacts in North America, in both cultured and wild trout populations [2, 3]. Whirling disease negatively influences the propagation and survival of many salmonid species over six continents, with particularly severe consequences for rainbow trout [4]. Despite significant advances, salmonid WD continues to pose a severe threat to both wild and farmed rainbow trout. For example, in 2016, the Canadian Food Inspection Agency (CFIA; Government of Canada) confirmed the first cases of salmonid WD in Canada in ten locations, including Banff National Park, Alberta [5]. Recent detections of WD have been reported in Alberta in 2017 and 2018.

Rainbow trout are the most susceptible salmonid species to *M. cerebralis*. The life-cycle of *M. cerebralis* alternates between salmonid fish and the oligochaete host *Tubifex tubifex* [6]. After the ingestion of *M. cerebralis* spores by *T. tubifex*, they multiply in the intestine and release triactinomyxon spores, which are infectious for salmonid fish. The ability to maintain *M. cerebralis* alternately in trout and oligochaetes proved essential for experimental studies on how the parasite recognizes and attaches to its fish host [7, 8]. During the development of *M. cerebralis* in the epidermis of rainbow trout, it appears as though some of the parasites are killed [9], possibly by humoral responses in the fish’s skin [2]. On the other hand, the parasites are sheltered from host immune reactions while migrating through peripheral nerves and the central nervous system (CNS) [9].

Differences in WD susceptibility between rainbow trout strains have been reported [10–13]. The German Hofer (HO) strain of *Oncorhynchus mykiss* is known to demonstrate resistance [4, 10]. Despite a number of studies, the reasons for the differences in susceptibility are not completely clear and the mechanism that conveys the varying levels of resistance among salmonid species remains largely unknown. Several gene expression studies have aimed at elucidating the mechanisms involved in these interactions [14–16]. The expression of two natural resistance-associated macrophage proteins (Nramp α and Nramp β) was investigated after infection with *M. cerebralis*. A decline in gene expression of Nramp α and Nramp β was observed in the susceptible rainbow trout strain at 14 and 40 days post-exposure (dpe) [17]. In a genome-wide expression profiling study, several genes were significantly upregulated in the skin of resistant and susceptible rainbow trout strains following exposure to *M. cerebralis*. Most of the annotated genes have known molecular functions and are involved in the interferon system [15]. The expression of innate immune response genes IL-1β, IFN-γ, IRF1 and iNOS was increased in one or both rainbow trout susceptible and resistant strains at various time points after exposure to *M. cerebralis*. The interferon-related genes, IFN-γ and IRF1, had constantly increased expression in the susceptible strain compared with the resistant strain. This was attributed to a less successful early immune response in the susceptible strain [18]. A further study used arginase-2 and inducible nitric oxide synthase (iNOS) genes as references to the alternative and classical pathway of macrophage activation. The expression of arginase-2 was differentially regulated in susceptible and resistant rainbow trout strains. The expression of iNOS, which is known to induce large amounts of nitric oxide in macrophages as a host defense mechanism and has a potential function in inflammation [19], was however, significantly elevated from 24 hours post-exposure (hpe) to 8 dpe in the susceptible strain and only at 8 dpe in the resistant strain. The susceptible rainbow trout strain is likely unable to mount an effective immune response against *M. cerebralis* [16]. As in mammals, the innate immunity in fish drives and modulates the adaptive immunity [20]. Yet, the interactions between *M. cerebralis* and cells of the salmonid immune system have not been investigated in detail [21].

In this study, the kinetics of local and systemic immune cell responses in resistant and susceptible rainbow trout strains was investigated to elucidate the cellular basis driving immune responses in rainbow trout strains against *M. cerebralis*, which can help to understand the mechanisms underlying the variation in resistance to WD.

## Methods

### Fish

Two rainbow trout strains, the German Hofer strain (HO), which has acquired a degree of resistance to WD, and the highly susceptible North American strain (TL) were used in this study. Specific-pathogen-free (SPF) rainbow trout HO and TL strains were reared in separate tanks in our wet laboratory at 14 ± 2 °C. Fish were maintained in a flow-through system supplied with UV-treated ground water and fed *ad libitum* with commercial trout feed until the start of the experiment.

### Infective triactinomyxon spores of *M. cerebralis*

The complete life-cycle of *M. cerebralis* is maintained in our laboratory at Vetmeduni Vienna [8, 22]. The cultures of *Tubifex tubifex* oligochaetes maintained at 14 °C were infected with *M. cerebralis* spores isolated from laboratory-infected rainbow trout. The waterborne triactinomyxon stages (TAMs) were harvested using a 20-μm polyamide filter. Experimental conditions were maintained by filtering the TAMs (to monitor released TAMs and assess the required quantity for the exposure experiment) twice weekly.

### Experimental infection of rainbow trout and collection of samples

SPF rainbow trout (3–4 cm, 90 days-old) of each strain (*n* = 120) were distributed in replicate tanks (*n* = 2) each containing an equal number of fish (*n* = 60). Rainbow trout were exposed to freshly filtered triactinomyxon spores (1000 TAMs per fish for 1 h without water flow). Then fish were transferred into separate aquaria 1 hpe, receiving 15 °C water and fed daily with commercial trout diet. An equal number (*n* = 60) of non-exposed rainbow trout of each strain were kept under the same conditions as a negative control. Fish from both exposed and non-exposed groups were fed at 1% body weight per day. At 2, 4, 8, 12, 24 and 48 hpe, and 4, 8, 14 and 20 dpe, five fish from each group were euthanized with a dose of 0.05% (w/v) MS-222 anesthetic and dissected. The caudal fin (CF), head kidney (HK) and spleen (SP) were sampled to monitor the immune response assembled against *M. cerebralis*: CF as the target site of infection and local response, and HK and SP to evaluate the systemic response triggered. Leukocytes were then isolated from sampled tissues to monitor the kinetics of different cell populations by flow cytometry.

### Histological examination

To investigate whether parasite incidence in CF tissues correlates with parasite load and immune reactions in HO and TL, parts of the fins were collected between 2 hpe and 4 dpe. Since *M. cerebralis* migrates within 48 hpe to 4 dpe through peripheral nerves and the CNS to reach the head cartilage [9, 18], later time points were excluded. The collected fins were subjected to routine processing and paraffin embedding for histopathological evaluation. Tissue sections of 5 µm thickness were prepared, deparaffinized and dehydrated in graded series of ethanol (from ≥ 99.8 to 50%). The sections were stained with hematoxylin and eosin (H&E).

### DNA extraction and pathogen load

The severity of *M. cerebralis* infection at all time points was evaluated by assessing the parasite load using a TaqMan quantitative polymerase chain reaction (qPCR) assay. The genomic DNA was extracted from CF tissues using a DNeasy blood and tissue kit according to the manufacturer’s instructions (Qiagen, Hilden, Germany). The TaqMan assay was applied to amplify the *18S* rDNA of *M. cerebralis* and host reference insulin growth factor-I genes [23]. Parasite load for each individual represents the number of *M. cerebralis 18S* rDNA gene copies per 10^6^ rainbow trout cells. The qPCR was performed on a CFX96 Touch Real-Time PCR detection system (Bio-Rad, München, Germany).

### Flow cytometry and determination of the kinetics of the cellular response

Flow cytometry was performed to identify the most important cell types participating in the immune reaction and to determine changes in the immune cell composition in response to *M. cerebralis* infection in the two rainbow trout strains. Forward and side scatter gating were used to exclude debris and identify cell populations based on size and granularity. Forward scatter (FSC) indicates cell size, whereas side scatter (SSC) is related to cell complexity (granularity). The small leukocytes with low granularity and small size, located FSC^low^; SSC^low^ are identified as lymphocytes. The bigger cells with higher granularity and bigger size, sited FSC^high^; SSC^high^ are recognized as myeloid cells. Leukocyte populations were isolated from sampled tissues (CF, HK and SP), stained with a set of monoclonal antibodies against myeloid cells, IgM heavy chain, CD8α and T cells [24, 25] and measured in the cytometer FACSCanto II (BD Biosciences, San José, USA).

### Sampling and leukocyte preparation

The organs (CF, HK and SP) were isolated from five fish from each group and used to prepare single cell suspensions. HK and SP tissues were disaggregated in Dulbecco’s phosphate-buffered saline (DPBS; Sigma-Aldrich, Vienna, Austria) 2% NCS (FACS buffer, FB) in a Corning^®^ cell strainer and then carefully layered onto 3 ml of Percoll (Sigma-Aldrich) in a 15 ml conical centrifuge tube, then centrifuged (with brake level 4) at 510×*g* for 30 min at 4 °C. The leukocytes were collected from the Percoll interface, washed once with FB and counted in a Neubauer chamber. Caudal fins were washed with 5 ml of DPBS containing 1 mM EDTA and 1 mM β-mercaptoethanol for 20 min in a shaking agitator at 5 °C. Then, the samples were washed 3 times with DPBS. The pre-treated fins were placed in new Petri plates and cut into small pieces, adding 0.15 mg/ml collagenase (Sigma-Aldrich) in Leibovitz L-15 media (Sigma-Aldrich). The digestion was performed for 30 min at room temperature with soft agitation. The supernatant was recovered and the pieces washed with FB and passed through a cell strainer. Samples were centrifuged at 510×*g* at 4 °C for 6 min; the cells were resuspended in 1 ml of FB and counted.

### FACS analysis

During all staining steps, the cells were maintained on ice and secondary antibodies were covered to protect them from light. The leukocytes were prepared as above and stained as follows. A first blocking step was made prior to antibody staining by incubating 5 × 10^5^ cells per each tube with FB for 20 min at 4 °C. After the primary antibodies were added to the cells and incubated for 20 min at 4 °C (Table 1), samples were washed with 700 µl of FB and centrifuged at 510×*g* for 6 min at 4 °C. The supernatant was discarded and the cells were incubated with fluorochrome-labeled secondary antibodies for 20 min at 4 °C (Table 1). A washing step was performed as above. Auto fluorescence and secondary antibodies tubes controls were also included. The viability of the cells was assessed prior the measuring by incubating with 1 µg of propidium iodide (Sigma-Aldrich). Finally, the samples were measured on a FACS Canto II cytometer and analysed by BD FACSDIVA software (BD Biosciences). Cell doublets and dead cells were excluded from the analyses.

**Table 1:** Monoclonal antibodies and fluorochromes used in this study for staining of the CF, HK and SP leukocyte subpopulations

Monoclonal antibodies	Stained cells	Fluorochrome
MAb21	Myeloid cells	Alexa Fluor 488
MAb1.14 and MAbN2	IgM^+^ B cells	Alexa Fluor 488
MAbD30	T lymphocytes	Alexa Fluor 405
MAbCD8α	CD8^+^ T cells	Alexa Fluor 647

### Statistical analysis

The differences in the parasite load for each group were analysed by a general linear model with repeated measurements and Sidak’s procedure. One-way ANOVA with Tukey’s α-correction was used to examine the differences of parasite load between HO and TL at each time point. To identify significance in the flow cytometric data between exposed and control fish of each strain, a non-parametric Mann-Whitney U-test was used. Differences between exposed and control fish were tested for significance between strains, also using a Mann-Whitney U-test. For both within and between strain comparisons, *P-*values < 0.05 were considered significant.

## Results

### *Myxobolus cerebralis* infection prevalence and parasite intensity in caudal fin during whirling disease development

**Fig. 1:**
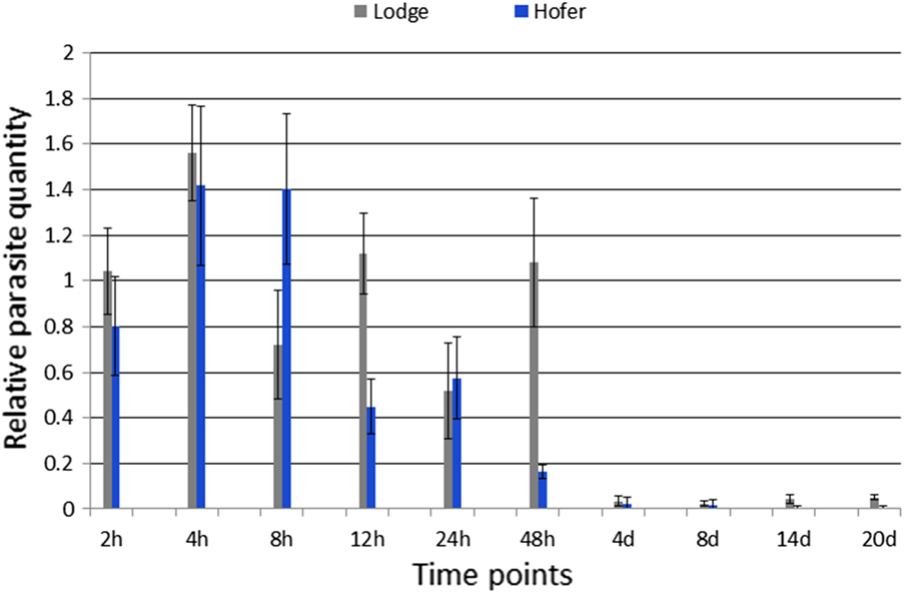
Pathogen intensity in caudal fin tissues of susceptible Trout Lodge (TL) and resistant HO (German) rainbow trout strains after *Myxobolus cerebralis* exposure. Forty-eight hours post-exposure, the HO strain has significantly reduced pathogen levels compared to the TL strain. At 4 dpe most *M. cerebralis* have left the skin tissue and are no longer detectable in fins

Exposure of TL and HO to *M. cerebralis* resulted in an infection prevalence of 100% (10/10 fish sampled) as assessed by qPCR. The parasite load in CF samples was higher in the TL strain at nearly all time points when compared with the HO strain. At 2 hpe, the parasite load in TL was not significantly increased relative to HO. Both strains exhibited their highest parasite intensity values at 4 hpe (Fig. 1). By 12 hpe, the parasite load of HO decreased 3.5-fold (*F*_(9,40)_ = 5342.330, *P* = *<* 0.0001) compared to its highest value at 4 hpe. The parasite intensity of TL also decreased between 4 and 12 hpe; however, this decline was more modest (< 1-fold). The HO strain exhibited a major reduction of parasite intensity at 48 hpe (> 7-fold) compared to 4 hpe value, while TL had > 5-fold parasite intensity compared to HO. At all time points between 2 and 48 hpe, with the exception of 8 hpe and 24 hpe, the HO strain had lower parasite intensity than the TL strain. At 4 dpe, parasite load was significantly reduced in both rainbow trout strains, likely because most remaining developmental stages cells have entered the nervous tissues, consistent with previous studies [9, 18]. The parasite was not detected in control samples for each strain.

### Histological assessment

**Fig. 2:**
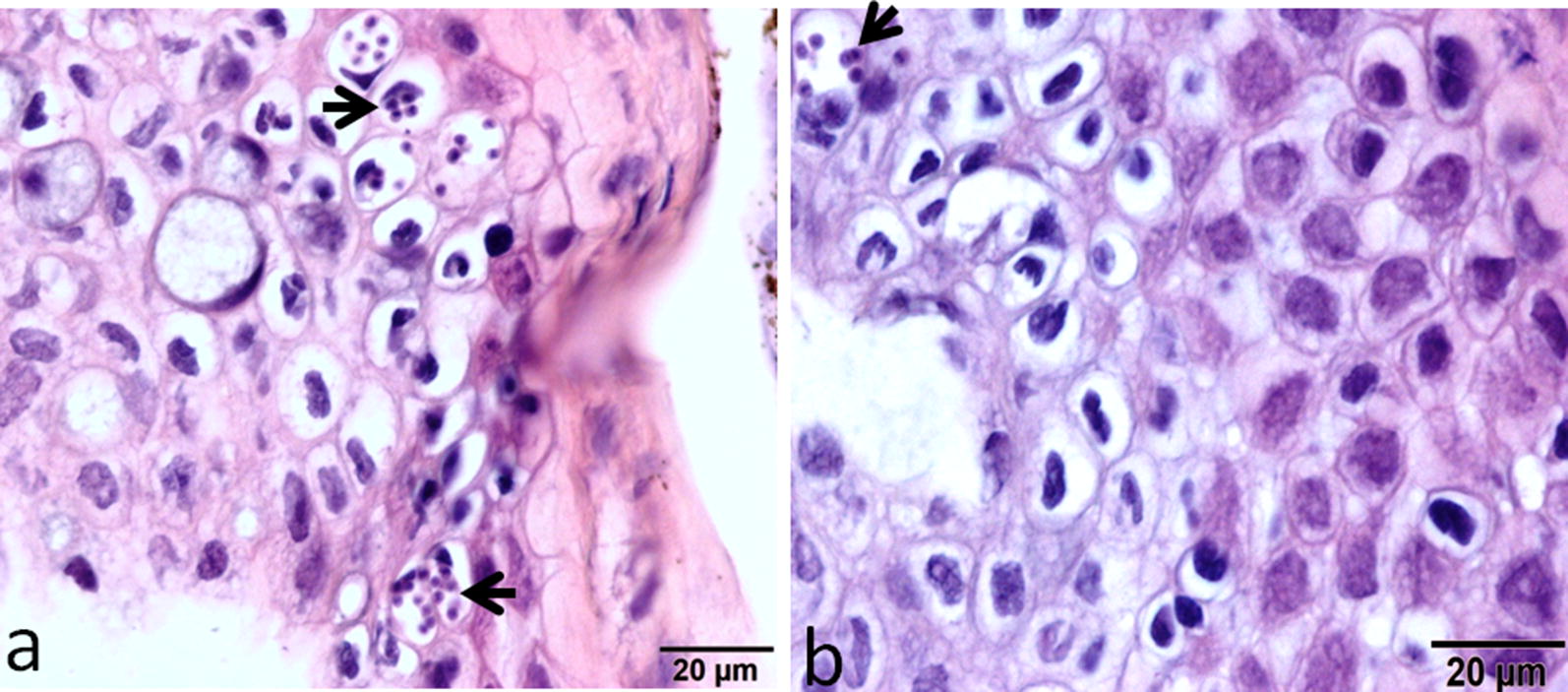
The figure shows numerous intracellular aggregates of the developmental stages of *M. cerebralis* detected in TL epidermis at 12 hpe (**a**), while in the HO strain, fewer parasite stages were observed in the epidermis at 12 hpe (**b**)

More parasite stages of *M. cerebralis* were detected in the epidermis of the TL strain at 12 hpe and intracellular aggregates of dark stained developmental stages were seen between the epithelial cells in the dermal layer (Fig. 2a). In the HO strain, parasite aggregates were lower in number than the parasites seen in TL strain (Fig. 2b).

### Flow cytometric analysis of main leukocytes from the caudal fin, head kidney and spleen

**Fig. 3:**
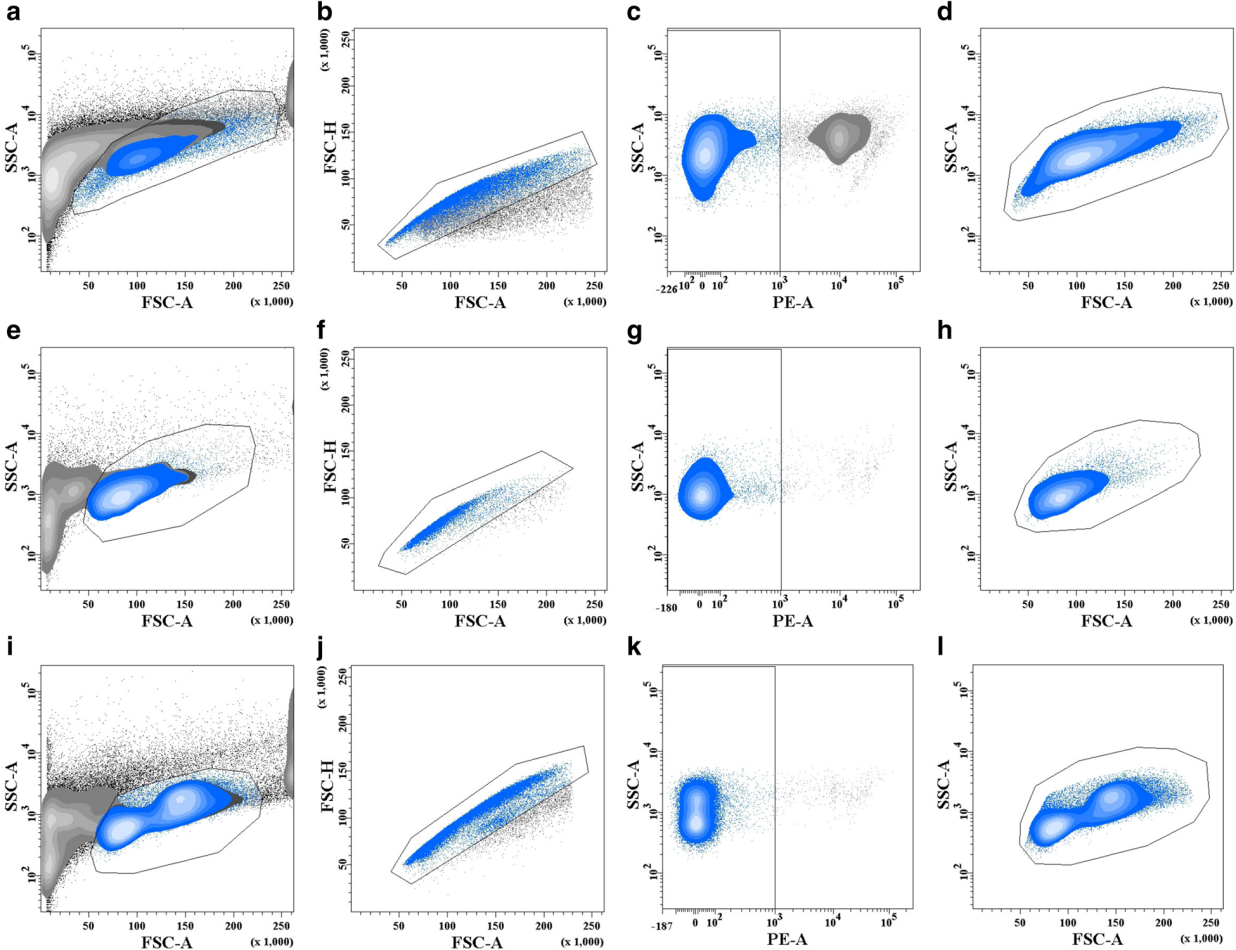
Gating of leukocyte subpopulations by flow cytometry. The figure shows forward *versus* sideward scatter plots of caudal fin (**a**–**d**), spleen (**e**–**h**), head kidney (**i**–**l**) tissues. The small leukocytes with low granularity are located FSC^low^; SSC^low^ and are identified as lymphocytes. The bigger cells with a higher granularity are found FSC^high^; SSC^high^ and recognized as myeloid cells

Flow cytometric analysis of leukocytes from CF tissue (Fig. 3a–d) revealed a cell population composition similar to that analyzed from the rainbow trout major lymphoid organs, i.e. SP (Fig. 3e–h) and HK (Fig. 3i–l). Using light scattering properties FSC (size) and SSC (granularity), the major types of leukocytes were distinguished (Fig. 3). The small leukocytes with low granularity (FSC^low^; SSC^low^) were identified as lymphocytes. The bigger cells with a higher granularity were recognized as myeloid cells (FSC^high^; SSC^high^). Very small events (FSC^low low^) corresponding to cell debris were excluded from the analysis; additionally, very big events (FSC^high high^) were also excluded as they represent cell aggregations. Both gating criteria were incorporated to avoid false positive signals. MAb1.14 and MAbN2 recognised the heavy and a light chain of IgM^+^ B cells as previously reported [24]. The number of IgT^+^ mucosal B cells could not be assessed due to the limited availability of specific monoclonal antibodies [26]. Within the T cells population, CD8^+^ T cells were recognized using anti-CD8α MAb. All T cells except CD8^+^ T cells were defined as CD8^−^ T cells likely CD4^+^ T helper cells [25–28].

### Flow cytometric analysis of caudal fin

**Fig. 4:**
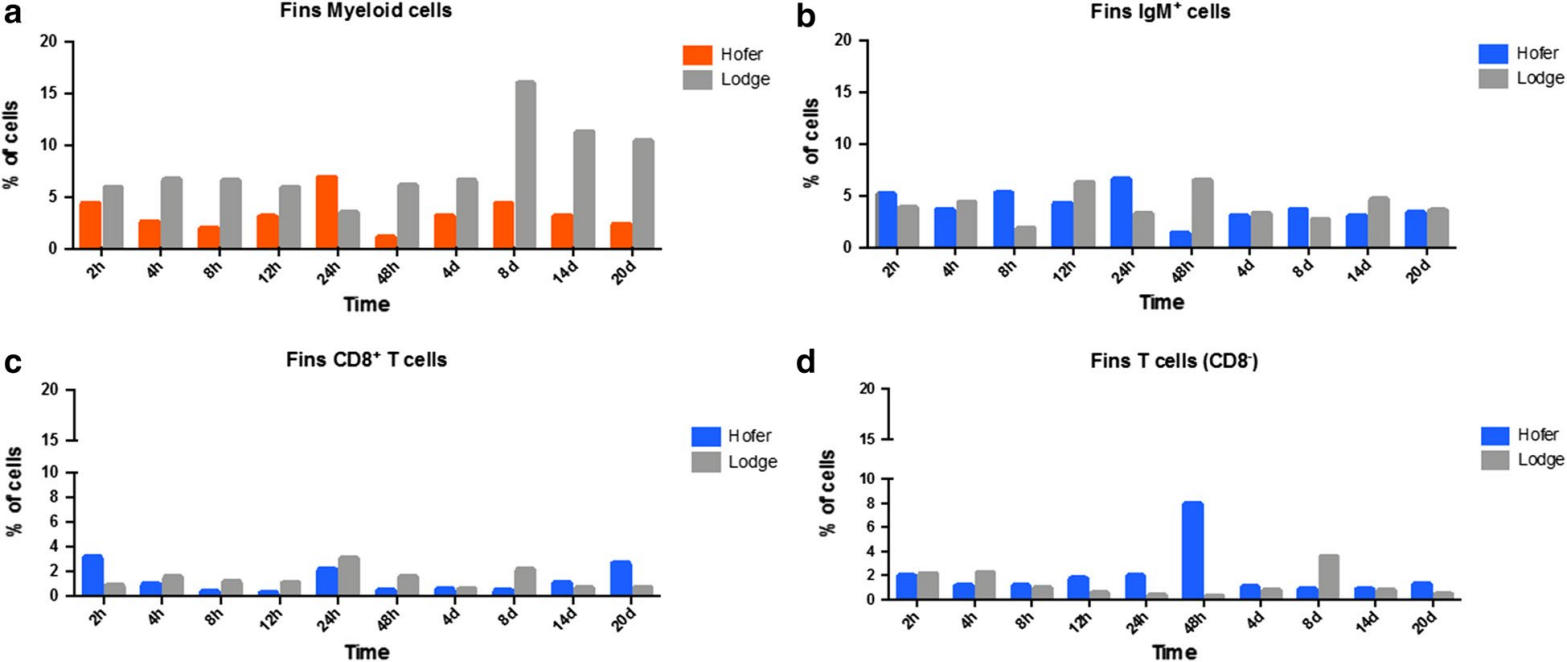
Flow cytometric analysis of HO and TL CF myeloid cells (**a**), IgM^+^ B cells (**b**), CD8^+^ T cells (**c**) and CD8^−^ T cells (**d**)

Flow cytometry analysis of TL CF leukocytes showed significant increases of myeloid cells (*U* = 9, *Z* = − 3.061, *P* = 0.002) relative to HO at the almost all time points except for 24 hpe in the HO strain (Fig. 4a). Major significant increases of IgM^+^ B cells (*U* = 0, *Z* = − 2.506, *P* = 0.012) occurred in HO at 8 and 24 hpe, while in TL increases were observed at 12 and 48 hpe (Fig. 4b). In HO, flow cytometry analysis demonstrated a broad decrease of larger granular cells of the myeloid origin (Fig. 4a) and a general increase of T cells (Fig. 4c, d). Significant increases (*U* = 0, *Z* = − 2.506, *P* = 0.012) of CD8^+^ T cells were observed in HO at 2 hpe, and at 14 and 20 dpe, as well as at 6 time points in the TL strain, i.e. at 4, 8, 12, 24 and 48 hpe and at 8 dpe (Fig. 4c). Significant increases of CD8^−^ T cells (*U* = 0, *Z* = − 2.506, *P* = 0.012) were observed in HO at 12 and 24 hpe; the highest elevation (> 9-fold) was detected at 48 hpe when compared with TL (Fig. 4d).

### Flow cytometric analysis of head kidney leukocytes

**Fig. 5:**
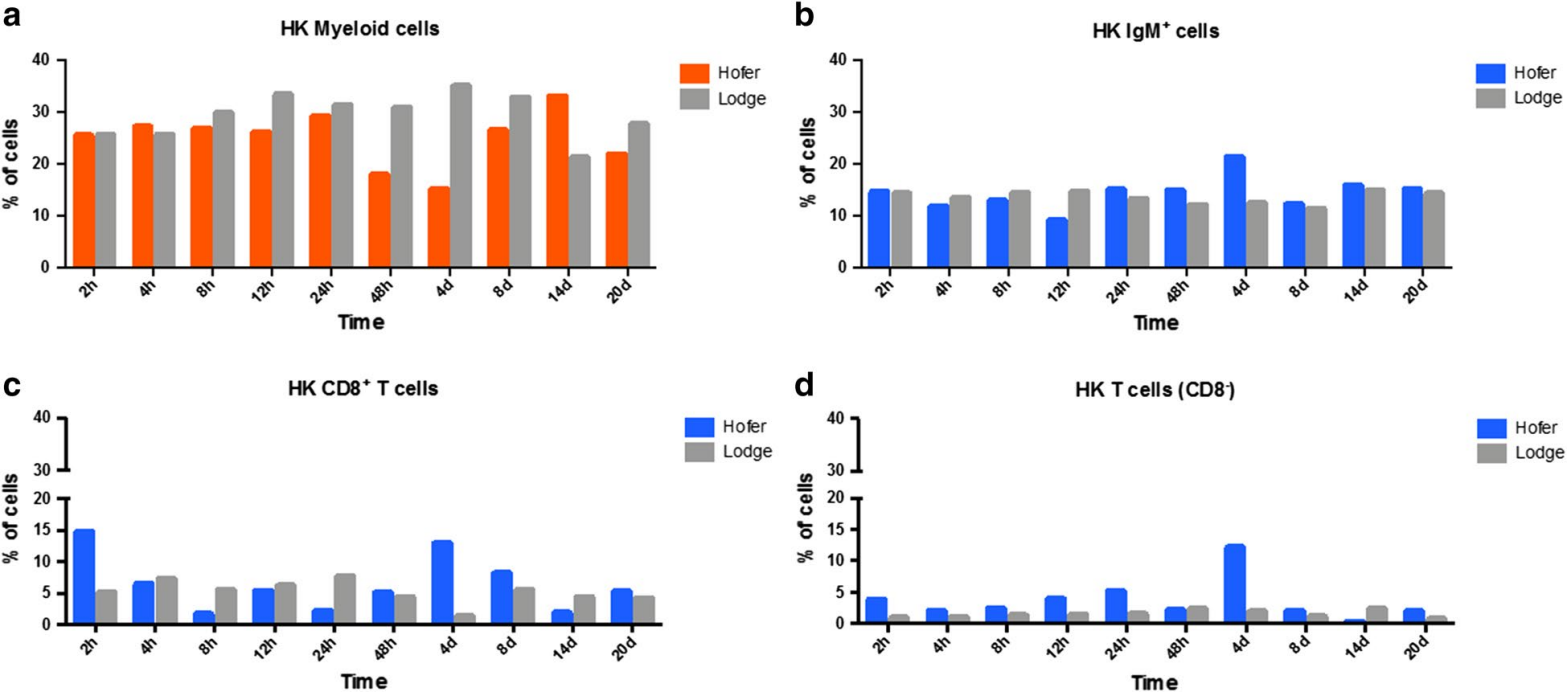
Flow cytometric analysis of HO and TL HK myeloid cells (**a**), IgM^+^ B cells (**b**), CD8^+^ T cells (**c**) and CD8^−^ T cells (**d**)

Flow cytometry of the HO HK leukocytes demonstrated significant increases of T cells and decreasing larger granular cells of the myeloid origin compared with TL (Fig. 5a–d). In TL, myeloid cells increases were observed at nearly all time points; however, these were only significant at 48 hpe and 4 dpe compared with HO (Fig. 5a). There was a significant increase of IgM^+^ B cells (*U* = 0, *Z* = − 2.506, *P* = 0.012) in HO compared to TL at 4 dpe (Fig. 5b). Additionally, significant increases (*U* = 0, *Z* = − 2.506, *P* = 0.012) were observed in CD8^+^ T cells at 2 hpe and 4 dpe in HO and at 24 hpe in TL (Fig. 5c). CD8^−^ T cells were significantly increased (*U* = 18.5, *Z* = 2.343, *P* = 0.019) at almost all time points in the HO except for 14 dpe (Fig. 5d).

### Flow cytometric analysis of spleen leukocytes

**Fig. 6:**
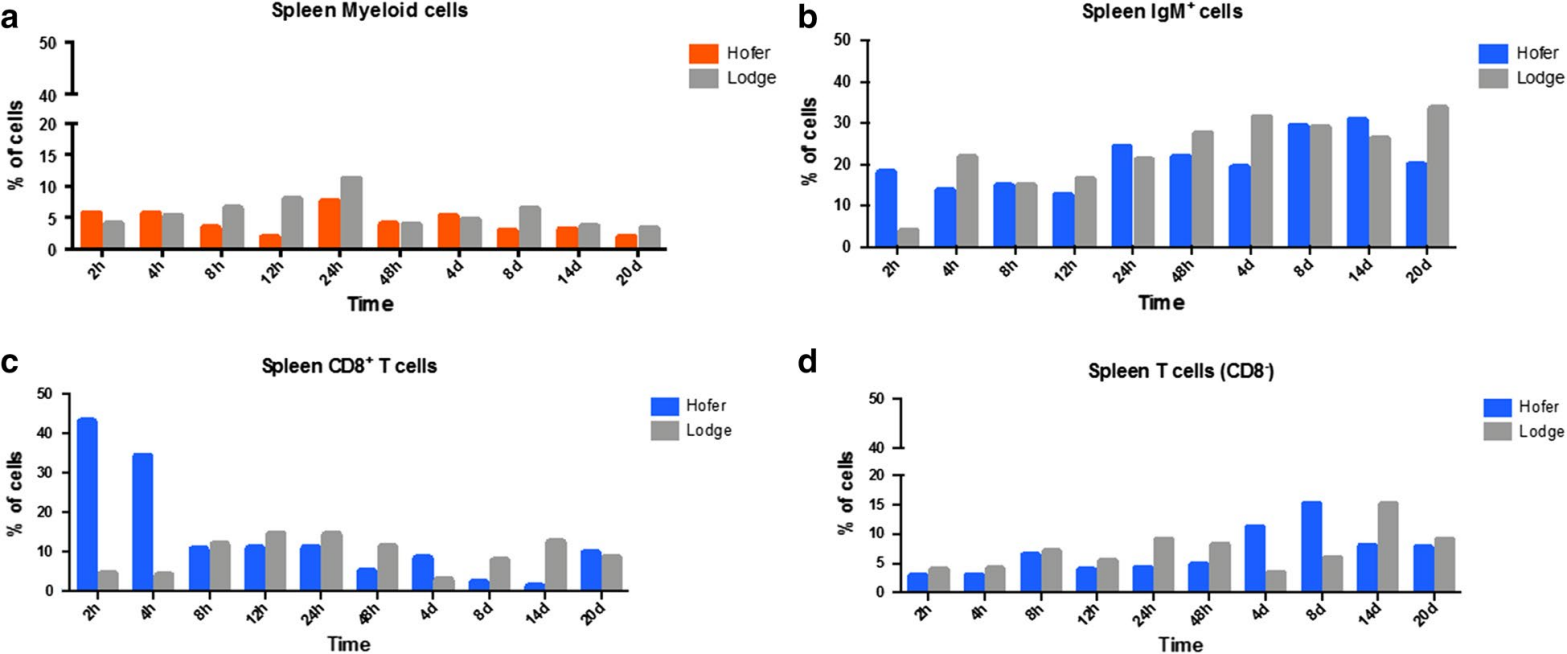
Flow cytometric analysis of HO and TL HK myeloid cells (**a**), IgM^+^ B cells (**b**), CD8^+^ T cells (**c**) and CD8^−^ T cells (**d**)

Flow cytometry of the SP leukocytes of HO showed significant increases of T cells at multiple time points and decreasing larger granular cells of the myeloid origin (Fig. 6a–d). In TL, myeloid cells increase was observed at nearly all time points except for 2 hpe (Fig. 6a). The proportion of IgM^+^ B cells increased in the HO strain significantly (*U* = 0, *Z* = − 2.506, *P* = 0.012) at 2 hpe and there were non-significant increases at 24 hpe and 14 dpe, while in TL increases were observed at 4, 12, 48 hpe and 4 and 20 dpe (Fig. 6b). There were prominent increases in CD8^+^ T cells at 2 and 4 hpe (> 9-fold) in HO (Fig. 6c). Significant increases of CD8^−^ T cells (*U* = 0, *Z* = − 2.506, *P* = 0.012) were observed in HO at 4 and 8 dpe, and at 24 hpe and 14 dpe in TL (Fig. 6d).

## Discussion

Despite recent progress, knowledge about immune response mechanisms able to neutralize the infection of *Myxobolus cerebralis* and avert its harmful effects is still limited. Differences in WD susceptibility exist between rainbow trout strains [10, 11]. Reasons for the differences in susceptibility are not completely clear and the mechanism that conveys the varying levels of resistance to WD remains largely unknown. Parasite stages migrate to the peripheral nerves within two to four days and during development of *M. cerebralis* in trout epidermis, some of the parasite stages are destroyed and parasitic stages in the subcutis are surrounded by host immune cells [9]. Thus, early interactions within the skin are likely crucial in determining why certain salmonid species are more resistant than others [6]. In this study, the kinetics of local and systemic immune cell responses to *M. cerebralis* early invasion was explored in two rainbow trout strains, the susceptible TL and the more resistant HO, to explore the cellular basis for WD susceptibility and resistance.

To our knowledge, this is the first time that a flow cytometry analysis was performed with leukocytes extracted from the fins of the fish, allowing a proper analysis of immune cell composition under a parasitic infection. The results of this study indicate that the immune response to *M. cerebralis* is generally evident and more powerful in the susceptible strain at almost all time points, involving every major cellular component of the innate immune system, especially cells of myeloid origin. The overwhelming and excessive immune response in TL likely leads to irreversible inflammatory reactions and tissue destruction evoked and boosted by parasite development thereby increasing host vulnerability.

Resistant fish showed strong immune responses to *M. cerebralis* at 2, 4, 12 and 48 hpe and at 4 dpe, characterised by significant increases of CD8^+^ and CD8^−^ T cells in the CF, HK and SP.

Indeed, HO demonstrates appropriate protection against the parasite as confirmed by parasite load and histology sections. In HO, the lowest parasite load at 48 hpe implies a successful parasite clearance and indicates effective immune response. This correlates with the highest level for CD8^−^ T (likely CD4^+^) cells observed at the same time point suggesting the involvement of this subpopulation in the resistance mechanisms of the HO strain against *M. cerebralis*. Previous gene expression studies showed that the expression levels of various innate immune response genes were higher in susceptible rainbow trout. This was attributed to less effective immune response. A dramatic upregulation of transcripts relating to the innate immunity was observed in susceptible fish but this did not provide protection. On the other hand, the resistant strain was characterized by a lower inflammatory response [16, 18]. In the present study, the inability of the susceptible TL strain to limit the infection likely resulted in the induction of an uncontrolled immune response involving a high representation of myeloid cells in CF, HK and SP, leading to subsequent inflammatory reactions, while more controlled immune cell responses are evident in the resistant HO strain. Myxozoan parasites can modulate the pro-inflammatory cellular responses including phagocytosis, oxidative phagocytic activity and complement activity [29]. While inflammation is crucial to the efficiency of the innate immune response, long-term activation of inflammatory processes can be seriously detrimental to the host and indicates a less effective immune response [16, 18, 30]. In fact, a lower inflammatory response combined with an adaptive T-cell response determined resistance and effectively enhanced viral (infectious salmon anemia virus) clearance and survival of challenged Atlantic salmon [31]. Here, the flow cytometry indicates significant increases of major immune cells in the susceptible TL strain. On the other hand, significant increases of T cells and major decreases of larger granular myeloid cells are characteristics of the resistant HO strain.

Kinetics of immune cell responses demonstrates that while a clear cellular immune response is observed in both resistant and susceptible strains, there are prominent differences between the two phenotypes. The susceptible TL strain showed marked upregulation of myeloid cells that evidently failed to protect against the parasite. In contrast, the resistant fish demonstrated a less pronounced immune response involving upregulation of CD8^+^ and CD8^−^ (likely CD4^+^) T cells. Based on the parasite load and flow cytometry data, it appears that the HO strain elicits an innate immune response that provides effective protection, which is in agreement with the concept of a resistant strain overcomes the infection fast corresponding to the findings of previous gene expression studies [16, 18].

The initial time points correlate with *M. cerebralis* multiplying and migrating from the epidermis to the dermis until progressively migrating to peripheral nerves [2]. The tendency of decreased pathogen load in HO in comparison with TL largely persists until the majority of parasitic cells have either degenerated or entered nervous tissue within 2 to 4 dpe [9]. Interferon pathways primarily play a key role in host defense against invading pathogens. The expression of two interferon-related genes, IFN-γ (a pro-inflammatory cytokine involved in host defense) and IRF1 (a transcription factor that regulates expression of genes involved in IFN-γ signalling), was investigated [9]. The innate immune system of both strains was activated by 24 hpe, with the two interferon pathway genes IFN-γ and IRF1 upregulated to their highest levels. At 24 hpe and later time points, the susceptible strain drifted toward having superior upregulation of these genes than the resistant strain. This increased transcription is likely detrimental to the susceptible strain since IFN-γ expression must keep a balance between anti-pathogenic effects and host inflammatory tissue damage. During the later time points of early disease progression, the susceptible strain had constantly increased upregulation in comparison to the resistant strain [18]. Similarly, the increases of the main leukocyte populations observed in the present study do not correlate with increased resistance. Indeed, the increases of major leukocytes in TL were likely due to the inability of the susceptible fish to demonstrate an effective early immune response. Particularly, the observed leukocyte levels likely mirror increased parasite load of the susceptible strain rather than a protective outcome against WD. In fact, during evaluation of parasite load and FACS data, it was apparent that the increases of immune cells did not always correlate with increased resistance.

In the present study, the HO strain demonstrated increases of CD8^+^ and CD8^−^ (presumably CD4^+^) T cells. The biological network maintaining equilibrium between parasite clearance and tissue protection involves significant contribution from effector T cells other than Th1 and Th2, namely Th17 [32]. The characterization of the CD4^+^ T helper cell population Th17 has increased the complexity of host–parasite interactions, previously thought to be determined by Th1 or Th2 polarization [33]. In a previous study, the resistant strain showed high STAT3 expressions at various time points, although, STAT3 is not located in the revealed major effect WD quantitative trait locus (QTL) region explaining 50–86% of the phenotypic variation across multiple families [4, 34]. However, not being in the particular identified QTL region discovered so far does not exclude the likelihood that a candidate gene may contribute a causative role in host response to infection [18]. STAT3 becomes activated after phosphorylation of its tyrosine 705 in response to various ligands including IL-6 [35]. Higher STAT3 in HO likely endorse resistance through generation of a specific class of T helper cells [18]. STAT3 is critical for the differentiation of Th17 from naive CD4^+^ T cells [36]. A previous study found greater TGF-β expression for the HO strain in comparison to the TL strain in response to *M. cerebralis* exposure [14]. TGF-β is a cytokine with a key role in Th17 differentiation [35]. Th17 cells are characterized by their production of IL-17 playing critical roles in maintaining mucosal barrier, reducing pathogen load and contributing to pathogen clearance at mucosal barriers [37]. Additionally, Th17 functions as a link between innate and adaptive immunity [38, 39]. Th17 cells can modulate their differentiation program to produce either regulatory or pro-inflammatory cells [40, 41]. Regulatory Th17, known as Treg17 cells, are induced by IL-6 and TGF-β, while pro-inflammatory Th17 are induced by IL-23 and Il-1β. As TGF-β and IL-1β were differentially regulated in both strains [16–18], it would be informative to investigate the responsiveness of Th17 (pro-inflammatory) or Treg17 cells (regulatory) in both strains by investigating IL-23 and IL-6 in addition to IL-17 post-exposure to *M. cerebralis*. Developing more specific antibodies for subsets of T cells and T helper cells is necessary to allow further studies aimed at understanding the cellular immune response and elucidating mechanisms that underlie the differences in the salmonid resistance against *M. cerebralis*. However, gene expression studies using specific markers of various immune cells are required for confirmation and further examination of the cellular based immune response involved in WD resistance.

## Conclusions

This is the first flow cytometry analysis of leukocytes extracted from the fins demonstrating the modulation of fish immune cell composition to control or favour a parasitic infection at the portal of entry. The results from this study show that *M. cerebralis* stimulates leukocyte trafficking in rainbow trout. A local inflammatory response mediated by myeloid cells likely supports *M. cerebralis* migration to peripheral nerves while the systemic inflammatory response promotes further moving into the CNS and head cartilage leading to WD. In TL, the increasing number of CF leukocytes indicated a strong local cellular inflammatory response correlating with parasite load. The excessive immune reactions likely lead to subsequent tissue damage and support parasite invasion. In HO, CD8^+^ T cells responded within hours in the fins, and also in the HK and SP. In HO, CD8^−^ T cells including CD4^+^ T cells also increased. Finally, in HO, T lymphocytes likely drive the immune response and mediate resistance against *M. cerebralis*. While the resistant phenotype shows more effective cellular immune response, both resistant and susceptible fish can become infected with *M. cerebralis*. The susceptible TL strain is characterized by a much larger, yet ineffective, immune cell response, largely characterized by increases in myeloid cells. Resistant fish demonstrate a more moderate, putative T lymphocyte-mediated immune response, which may contribute to their resistance.

## Data Availability

All data supporting the findings of this study are presented in the article.

## Abbreviations

CF: caudal fin
CNS: central nervous system
dpe: days post-exposure
FSC: forward scatter
HK: head kidney
HO: German Hofer strain
hpe: hours post-exposure
qPCR: quantitative PCR
SP: spleen
SSC: side scatter
TAMs: triactinomyxons
TL: American Trout Lodge
WD: whirling disease
